# Thirty-Day High-Grade Aortic Valve Block Post-Transcatheter Aortic Valve Replacement in Patients Discharged on Heart Rhythm Monitor

**DOI:** 10.1016/j.shj.2024.100317

**Published:** 2024-05-22

**Authors:** Mohamad S. Alabdaljabar, Mohamed Elhadi, Rajiv Gulati, Charanjit S. Rihal, Paul A. Friedman, Yong-Mei Cha, Mackram F. Eleid

**Affiliations:** a Department of Internal Medicine, Mayo Clinic, Rochester, Minnesota; b Department of Cardiovascular Medicine, Mayo Clinic, Rochester, Minnesota

**Keywords:** Heart rhythm monitor, High grade AV block, MoMe, TAVR, Transcatheter aortic valve replacement

## Abstract

**Background:**

Conduction disease is an important and common complication post-transcatheter aortic valve replacement (TAVR). Previously, we developed a conduction disease risk stratification and management protocol post-TAVR. This study aims to evaluate high-grade aortic valve block (HAVB) incidence and risk factors in a large cohort undergoing ambulatory cardiac monitoring post-TAVR according to conduction risk grouping.

**Methods:**

This single-center, retrospective study evaluated all patients discharged on ambulatory cardiac monitoring between 2016 and 2021 and stratified them into 3 groups based on electrocardiogram predictors of HAVB risk (group 1 [low], group 2 [intermediate], and group 3 [high]). HAVB was defined as ≥2 consecutive nonconducted P waves in sinus rhythm or bradycardia <50 beats/minute with a fixed rate for atrial fibrillation/flutter. Descriptive statistics were used to show the incidence and timeline, while logistic regression was utilized to evaluate predictors of HAVB.

**Results:**

Five hundred twenty-eight patients were included (median age 80 years [74-85]; 43.8% female). Forty-one patients (7.8%) developed HAVB during ambulatory monitoring (68% were asymptomatic). Over a median follow-up of 2 years (1.3-2.7), the overall mortality rate was 15.0% (30-day mortality rate of 0.57%, n = 3). Risk factors for HAVB were male sex (odds ratio [OR] = 2.46, *p* = 0.02, 95% CI = 1.21-5.43), baseline right bundle branch block (OR = 2.80, *p* = 0.01, 95% CI = 1.17-6.19), and post-TAVR QRS >150 ​ms (OR = 2.16, *p* = 0.03, 95% CI = 1.01-4.40). The negative predictive value for patients in groups 1 and 2 for 30-day HAVB was 95.0 and 93.8%, respectively.

**Conclusions:**

The risk of 30-day HAVB post-TAVR on ambulatory monitoring post-TAVR varies according to post-TAVR electrocardiogram findings, and a 3-group algorithm effectively identifies groups with a low negative predictive value for HAVB.

## Highlights

• The timeline and symptoms of patients discharged on ambulatory monitoring post-transcatheter aortic valve replacement are not clear.
• The incidence of high-grade aortic valve block was 7.8% (68% asymptomatic).
• Eighty percent of high-grade aortic valve block events occurred in the first 2 ​weeks.
• These findings could help establish a national consensus on post-transcatheter aortic valve replacement conduction disease management.

## Graphical abstract

**Figure:**
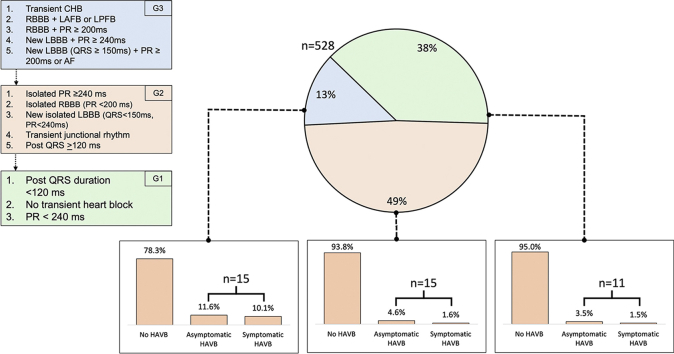

## Introduction

Transcatheter aortic valve replacement (TAVR) is a common treatment for patients with symptomatic aortic stenosis. One of the most common adverse complications of TAVR is conduction system disease and its sequelae. Although the rates of many TAVR complications have decreased over time with the advancement in TAVR device technology, techniques, and expertise, the rates of conduction disease post-TAVR remain high in many studies.^1^^,^^2^ The incidence of high-grade aortic valve block (HAVB) ranges between 12% and 36%, depending on the type of prosthetic valve used (lower in balloon expandable valve [BEV] compared to self-expanding valve [SEV])^3^ as well as the patient population (lower in low-risk TAVR populations). The rate of delayed HAVB (>48 ​hours post-TAVR) is estimated at 7% to 10%.^4^, ^5^, ^6^

Two previous studies assessed the rate of delayed HAVB within 30 days using ambulatory cardiac monitoring^4^^,^^5^ and showed comparable delayed HAVB rates (7%-10%). However, since implementation of a standardized conduction management algorithm designed to better identify and stratify patients according to the risk of progressive conduction disease, rates of undiagnosed/delayed HAVB are expected to decrease.^7^ The aim of this study was to assess the rate of delayed HAVB, symptoms, predictors of delayed HAVB, and all-cause mortality in a large series of patients undergoing TAVR who were discharged on a 30-day ambulatory cardiac monitor since the implementation of a standardized conduction management algorithm at our institution. We aimed to evaluate the 30-day outcomes in a high-volume TAVR center in patients discharged with ambulatory monitoring to evaluate the performance of the current approach and determine the optimal duration of ambulatory monitoring.

## Methods

### Study Patients

This study was approved by the Mayo Clinic Institutional Review Board. Since this is a retrospective observational study, informed consent was not required. The authors had full access to all data and took responsibility for its integrity. The electronic medical records of patients who had TAVR at Mayo Clinic (2012-2021) in Rochester, Minnesota were retrospectively evaluated, and baseline characteristics including transthoracic echocardiogram, and TAVR-related factors were retrieved and stored. Patients with prior permanent pacemaker (PPM)/implantable cardioverter-defibrillator, intraprocedural death, or conversion to surgical aortic valve (AV) replacement were excluded. Before TAVR, a multidisciplinary heart team evaluated all patients. TAVR was performed as previously described.^8^^,^^9^ All patients had continuous telemetry for at least 24 ​hours post-TAVR. Twelve-lead electrocardiogram (ECG) was obtained post-TAVR and daily thereafter until the day of discharge.

This study included only patients who were discharged on a 30-day cardiac monitor between 2016 and 2021. With the lack of national guidelines, an institutional protocol was developed through multidisciplinary collaboration to risk stratify patients based on baseline, procedural, and postprocedural factors, as previously described.^2^ In brief, patients were divided into 3 categories previously described based on risk of 30-day HAVB from post-TAVR ECG: group 1 (normal QRS duration or QRS <120, PR < 240 ​ms, and no transient HAVB event); group 2 (new left bundle branch block [LBBB] + PR < 240 ​ms + QRS <150 ​ms, isolated PR ≥ 240 ​ms, isolated right bundle branch block [RBBB] + PR < 200 ​ms, transient junctional rhythm, or nonspecific conduction disease with post-QRS >120 ​ms); and group 3 (transient HAVB, new LBBB + PR ≥ 240 ​ms, new LBBB + QRS ≥150 ​ms + PR ≥ 200 ​ms/incalculable PR, or RBBB with first degree AV block/left fascicular block).^7^ Patients who had PPM were excluded. Patients in groups 2 (mainly) and 3 were discharged on a remote 30-day continuous ambulatory ECG monitoring system (BodyGuardian; Preventice Technologies, Inc; Eagan, MN) or MoMe Kardia System (InfoBionic, Chelmsford, MA) to evaluate for late HAVB and the need for PPM implantation. Other patients who received 30-day heart monitoring that were not part of group 2 included patients who were discharged on a monitor before the development of the algorithm, and patients who were evaluated on a case-by-case basis by the electrophysiology team, and a shared-decision was made to discharge patients on these monitors. All patients’ heart rhythms were continuously monitored; upon the development of HAVB, the treating team was immediately notified. Patients were then directly contacted, and a note was posted on their medical record documenting the time of event and presence/absence of symptoms.

### Primary Outcomes

The main cohort and results of this study are based on patients who were discharged from an ambulatory cardiac monitor (n = 528).

The primary endpoints were a) 30-day HAVB and b) 30-day mortality. Electrocardiographic evidence of HAVB was based on the presence of ≥2 consecutive nonconducted P waves for patients in sinus rhythm or bradycardia <50 beats/minute with a fixed rate for patients with atrial fibrillation/flutter. All patients’ medical records were reviewed for the development of symptoms at the time of HAVB detection.

### Statistical Analysis

Descriptive statistics were used for the analysis of baseline characteristics including pre- and post-ECG and TAVR-related parameters, with continuous data being reported as mean ± SD or median and interquartile range and categorical data being reported as a number and frequency (%). Continuous variables were described and evaluated for statistical significance using the independent *t* test, whereas continuous variables not normally distributed were analyzed using nonparametric Wilcoxon test, and categorical variables were assessed for significance using chi-square method. Logistic regression analysis was used to evaluate risk factors associated with the development of 30-day HAVB. Kaplan-Meier and Cox regression analyses were utilized to evaluate all-cause mortality. The number of variables in the multivariable model in the full group was limited to be less than the number of events/10 (to avoid model overfitting). The results of the logistic regression analyses were reported in terms of odds ratio (OR), 95% CIs, and *p* value. A statistical significance level of *p* < 0.05 was used. All data were exported and managed using Microsoft Excel. Statistical analyses were performed using BlueSky Statistics LLC, v10.2.0 (Chicago, IL).

## Results

### Baseline, Procedural, and Post-ECG Features

A total of 528 patients were included. The median age was 80 years (74-85) with 43.8% females (Table 1). The most common comorbid condition was hypertension (88%), followed by coronary artery disease (CAD) (61.0%). The median New York Heart Association class was 3 (2-3). The aortic valve area median was 0.87 (0.71-0.94) cm^2^, with an AV mean gradient of 46 (39-61) mmHg and a left ventricular ejection fraction of 63% (57-66). Most patients had the TAVR done under moderate sedation (87.5%), and the majority received BEV (93%) as opposed to SEV (7%). More than 90% of the valves used were SAPIEN 3 (Edwards Lifesciences, Irvine, CA). The most common valve diameter used was 26 mm (38.8%), with the most commonly used access being the femoral site (96.6%).

**Table 1:** Baseline characteristics

Parameter	Median (IQR) or frequency (%)
Age (y)	80 (74-85)
Female	43.80%
BMI (Kg/m^2^)	29.3 (25.6-33.6)
Hypertension	87.90%
Diabetes mellitus	34.40%
Prior stroke/TIA	12.90%
Atrial fibrillation/flutter	34.80%
ESRD on dialysis	2.50%
Smoking	3.00%
CAD	61.00%
Prior PCI	36.70%
Prior CABG	16.10%
STS score (%)	4.0 (2.3-5.6)
NYHA	3 (2-3)
AV area (cm2)	0.87 (0.71-0.94)
AV mean gradient (mmHg)	46 (39-61)
LVEF (%)	63 (57-66)
CAD (number of vessels)	
1	17.40%
2	17.00%
3	26.50%
Left main disease	9.7%
General anesthesia	12.50%
Moderate sedation	87.50%
Valve in valve	5.70%
Valve (mechanism)	
Self-expanding	7.00%
Balloon-expandable	93.00%
Valve type	
Corevalve	0.40%
Evolut PRO	0.40%
Evolut PRO PLUS	0.60%
Evolut PRO System	0.20%
Evolut Pro	1.10%
Evolut Pro Plus	1.70%
Evolut R	2.70%
Sapien 3	51.90%
Sapien 3 Ultra	41.10%
Valve diameter (mm)	
20	1.70%
23	29.70%
26	38.80%
29	27.70%
34	1.90%
35	0.20%
Access site	
Axillary	0.60%
Carotid	0.20%
Femoral	66.50%
Femoral artery	29.90%
Subclavian	0.20%
Transapical	2.10%
Transcarotid	0.40%
Transcaval	0.20%

Abbreviations: AV, aortic valve; BMI, body mass index; CABG, coronary artery bypass graft; CAD, coronary artery disease; ESRD, end-stage renal disease; IQR, interquartile range; LVEF, left ventricular ejection fraction; NYHA, New York Heart Association; PCI, percutaneous coronary intervention; STS, Society of Thoracic Surgeons; TIA, transient ischemic attack.

Baseline and post-TAVR ECG parameters are summarized in Table 2. Eighty-two percent of patients had baseline sinus rhythm. One-quarter (25.7%) had baseline first-degree heart block, with a median PR interval of 180 mm (160-207) and QRS interval of 98 mm (88-114). The rates of LBBB and RBBB were 9.3% and 5.9%, respectively. In the catheterization lab, 4.9% of patients had transient HAVB. Post-TAVR, sinus rhythm (80.1%) and PR interval of 180 mm (160-207) were comparable to the baseline ECG; however, QRS interval increased to a median of 124 (96-144). Approximately one-third of patients developed new LBBB (31.1%), whereas only 2.3% developed new RBBB.

**Table 2:** ECG changes and outcomes

Parameter	Median (IQR) or frequency (%)
ECG baseline	
Sinus rhythm	81.8%
PR interval (ms)	180 (160-207)
First-degree heart block	25.7%
Isolated PR interval >240 ​ms	5.7%
QRS duration (ms)	98 (88-114)
QRS duration >150 (ms)	6.1%
LBBB	5.9%
RBBB	9.3%
LAFB	3.8%
LPFB	0.3%
ECG post-TAVR	
Intraprocedural HAVB	
Transient	4.9%
Persistent	0.0%
Sinus rhythm	80.1%
First-degree heart block	11.9%
PR interval (ms)	180 (160-207)
<200	60.4%
200-400	28.8%
>400	10.8%
QRS (ms)	124 (96-144)
QRS >150 ​ms	18.0%
New LBBB	31.1%
New RBBB	2.3%
New LAFB	5.4%
New LPFB	0.0%

Abbreviations: ECG, electrocardiogram; HAVB, high-grade atrioventricular block; IQR, interquartile range; LAFB, left anterior fascicular block; LBBB, left bundle branch block; LPFB, left posterior fascicular block; RBBB, right bundle branch block; TAVR, transcatheter aortic valve replacement.

Characteristics were similar between those who were discharged on monitor with those who were not (median age was 79.2 [74-85] vs. 79.3 [74-85] years [*p* = 0.81], and female gender was 43.8% vs. 41.4% [*p* = 0.33]). The most common prosthetic valve was Sapien 3 (51.8% vs. 38.5%), followed by Sapien 3 Ultra (41.2% vs. 38.2%), Sapien (0% vs. 7.7%), Evolut R (2.6% vs. 4.3%), Sapien XT (0% vs. 3.9%), and CoreValve (0.38% vs. 4.1%), *p* < 0.001. Evolut Pro and Pro Plus were used in less than 3% of each of these two groups.

### Thirty-Day HAVB and PPM Implantation

Out of the 528 patients monitored for 30 days post-TAVR, 41 patients (7.8%) developed HAVB with an 8% rate of PPM implantation (Table 3). Sixty-eight percent of patients were asymptomatic at the time of HAVB, whereas the rest had either syncopal or presyncopal symptoms. The baseline and post-TAVR features were compared between both (symptomatic and nonsymptomatic) groups, and the only statistically significant factor between the groups was found to be baseline RBBB (present in 42.9% of the symptomatic vs. 11.1% in asymptomatic patients, *p* = 0.02).

**Table 3:** HAVB, PPM implantation, and death rate post-TAVR

Parameter	Number (frequency, %)
Outcomes	
Deaths	79 (15.0%)
Deaths within 30 d	3 (0.57%)
HAVB within 30 d	41 (7.8%)
Asymptomatic	28 (68.3%)
Presyncope	6 (14.6%)
Syncope	6 (14.6%)
Death	1 (2.4%)
PPM placed∗	42 (8.0%)
Percentage pacing (%)	26.0 (3.5-92.3)

Abbreviations: HAVB, high-grade atrioventricular block; PPM, permanent pacemaker; TAVR, transcatheter aortic valve replacement.

∗ One patient received PPM for another indication and not for HAVB.

One patient was found deceased at home, and the time of death was correlated with the time of HAVB recorded on the heart monitor. Another patient died because of injuries related to a motor vehicle accident, and the time of the accident was correlated with the time of HAVB detected on the heart monitor. These two patients, who died potentially because of HAVB events, were further evaluated. The first patient was a 71-year-old female with baseline normal sinus rhythm, PR interval of 162 mm, and QRS interval of 106. Post-TAVR (Sapien 3U valve) ECG showed a PR interval of 184 mm, QRS interval of 150 mm and new LBBB. On the heart monitor, the patient had evidence of a complete heart block with a 21-second pause followed by asystole. The second patient was an 87-year-old male with a history of atrial fibrillation and percutaneous coronary intervention. Baseline ECG was normal sinus rhythm with PR interval of 134 mm, QRS interval of 72 mm, and no evidence of conduction disease. Post-TAVR (Sapien 3 valve), the ECG showed a PR interval of 134 mm and QRS interval of 72 mm. His heart monitor showed 38 pause events greater than 3 ​seconds. The longest pause was 13 ​seconds.

**Figure 1:**
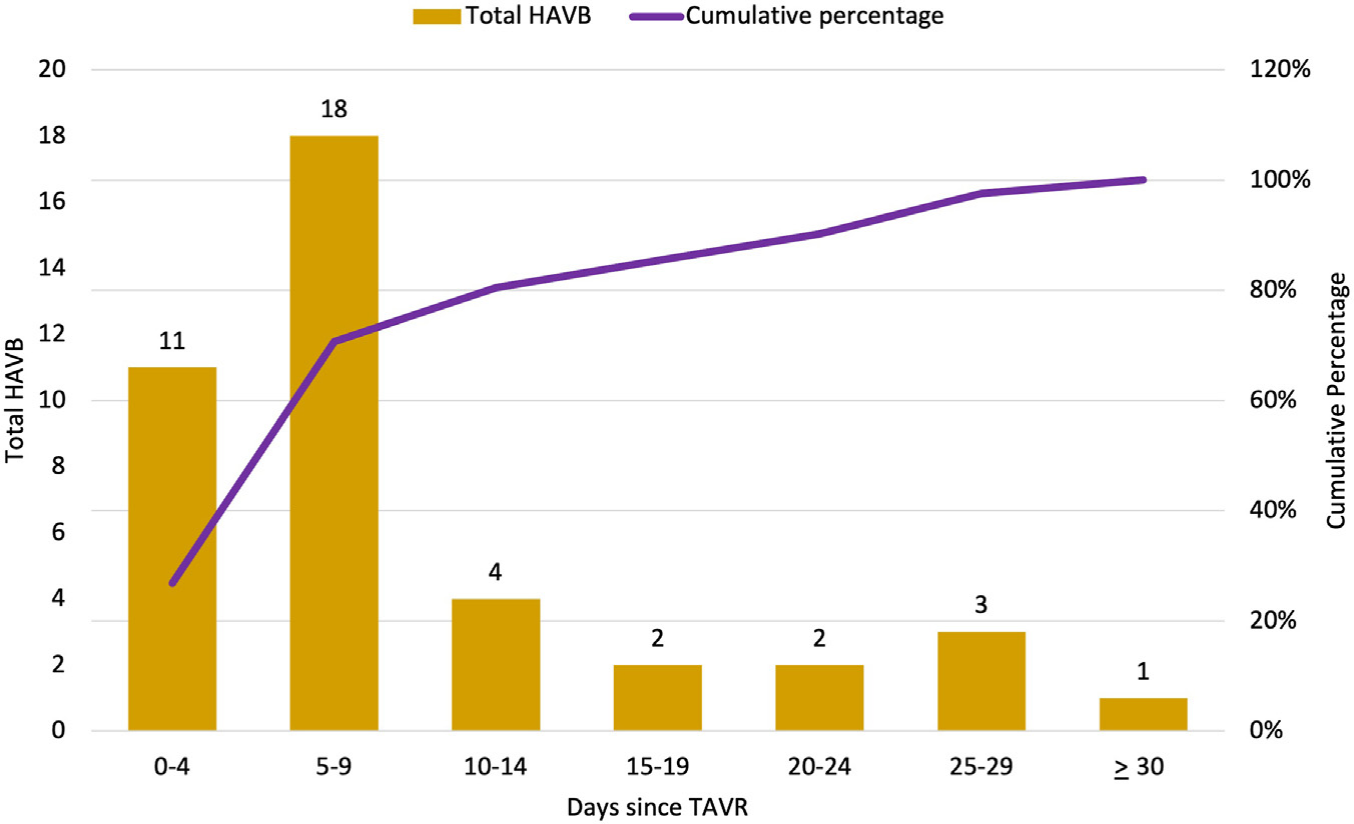
Bar chart with cumulative incidence showing timeline and frequency of HAVB development post-TAVR Abbreviations: HAVB, high-grade atrioventricular block; TAVR, transcatheter aortic valve replacement.

Graphical Abstract shows the breakdown of patients based on the assigned group and the percentages of patients who developed HAVB within 30 days. From each of these three groups, the rate of 30-day HAVB was highest in patients from group 3 (21.7%) and less than 7% in each of groups 1 and 2. The negative predictive value for patients in groups 1 and 2 to have 30-day HAVB was 95.0 and 93.8%, respectively. Figure 1 is a bar graph chart showing the frequency, incidence, and timing of HAVB. Most of the HAVB events occurred in the first 10 days (70%), and 80% of HAVB happened in the first 2 weeks post-TAVR. The current post-TAVR conduction management protocol used in our institution recommends patients in group 2 to be discharged on 30-day ambulatory cardiac monitor if patients meet at least one criterion of the post-TAVR ECG changes. The rate of 30-day HAVB based ECG changes in group 2 was as follows: 6.7% (isolated PR ≥ 240 ​ms), 0% (isolated RBBB + PR < 200 ​ms), 7.1% (new LBBB + PR < 240 ​ms + QRS <150 ​ms), 20% (transient junctional rhythm), and 6.0% (nonspecific conduction disease with post-QRS ≥120 ​ms).

### Sex-Related Differences

The rate of HAVB was analyzed based on sex; males were found to have a higher rate of HAVB than females (75.6 vs. 24.4%, *p* = 0.009). To better understand this, descriptive statistics showed that male patients had higher rates of diabetes mellitus, CAD, larger AV area, greater valve diameter, baseline PR and QRS intervals, and post-PR interval, yet they had a lower left ventricular ejection fraction and rate of new LBBB compared to female patients (*p* < 0.05). Differences between groups are detailed in Table 4.

**Table 4:** Clinical, ECG, and outcome differences based on sex

Median/percentage	Male	Female	*P* value
Age	80	81	0.34
HAVB within 30 d	10.4%	4.3%	0.01
PPM implantation	11.1%	3.9%	0.002
Death	15.8%	13.9%	0.53
BMI (Kg/cm^2^)	29.3	29.3	0.31
Hypertension	89.2%	86.1%	0.28
Diabetes mellitus	41.8%	27.3%	<0.001
ESRD on dialysis	3.4%	1.3%	0.13
Prior stroke/TIA	14.8%	10.4%	0.13
Atrial fibrillation/flutter	36.7%	32.5%	0.31
CAD	72.1%	46.8%	<0.001
NYHA	3	3	0.31
AV area	0.9	0.8	<0.001
AV mean gradient	46	45.5	0.76
LVEF (%)	61	64	<0.001
Valve diameter (mm)	26	23	<0.001
Baseline PR interval (ms)	194	168	<0.001
Baseline QRS interval (ms)	102	92	<0.001
Baseline LBBB	5.4%	6.5%	0.59
Baseline RBBB	10.8%	7.4%	0.18
Post-PR interval (ms)	202	182	<0.001
Post-QRS interval (ms)	120	124	0.31
New LBBB	26.3%	38.7%	0.003
New RBBB	3.0%	1.3%	0.19

Abbreviations: AV, aortic valve; BMI, body mass index; CAD, coronary artery disease; ECG, electrocardiogram; ESRD, end-stage renal disease; HAVB, high-grade atrioventricular block; LBBB, left bundle branch block; LVEF, left ventricular ejection fraction; NYHA, New York Heart Association; PPM, permanent pacemaker; RBBB, right bundle branch block; TIA, transient ischemic attack.

### Risk Factors for HAVB

A logistic regression multivariable model is shown in Table 5. The significant risk factors for 30-day HAVB in patients with ambulatory cardiac monitor were male sex (OR = 2.46, *p* = 0.02, 95% CI 1.21-5.43), baseline RBBB (OR = 2.80, *p* = 0.01, 95% CI 1.17-6.19), and post-QRS >150 mm (OR = 2.16, *p* = 0.03, 95% CI 1.01-4.40). For the baseline RBBB, 49 patients had evidence of baseline RBBB, nine of whom developed HAVB. Post-TAVR, 57 patients had RBBB, out of which the same nine patients with baseline RBBB were the ones who developed HAVB. All nine patients were in group 3 (eight with PR ≥ 200 and one with left anterior fascicular block). The post-TAVR QRS cutoff of 150 ​ms was chosen after examining different thresholds, and 150 ​ms was found to represent the best cutoff of increased risk for HAVB. Multivariable model accuracy was 0.92 with an area under the ROC curve of 0.67.

**Table 5:** Logistic regression showing predictors of HAVB within 30 d from TAVR

	Univariable				Multivariable			
	OR	*p* value	2.50%	97.50%	OR	*p* value	2.50%	97.50%
(Intercept)					0.03	<0.001	0.02	0.06
Age	0.98	0.45	0.94	1.03				
Male	2.58	0.01	1.28	5.64	2.46	0.02	1.21	5.43
Hypertension	0.99	0.99	0.38	2.63				
Diabetes mellitus	1.06	0.87	0.55	2.05				
ESRD on dialysis	0.99	0.99	0.13	7.81				
Prior stroke/TIA	0.94	0.89	0.35	2.47				
Atrial fibrillation/flutter	1.88	0.054	0.99	3.56				
CAD	2.09	0.05	1.00	4.36				
NYHA	0.93	0.78	0.56	1.54				
AV area	0.76	0.71	0.18	3.27				
AV mean gradient	0.99	0.47	0.98	1.01				
LVEF (%)	0.98	0.09	0.96	1.00				
Valve diameter (mm)	1.12	0.054	1.00	1.26				
Baseline PR interval (ms)	1.01	0.002	1.00	1.02				
Baseline QRS interval (ms)	1.01	0.06	1.00	1.02				
Baseline LBBB	0.81	0.78	0.19	3.52				
Baseline RBBB	3.14	0.01	1.33	6.82	2.80	0.01	1.17	6.19
Post-PR (ms)	1.01	0.003	1.00	1.02				
Post-QRS >150 (ms)	2.27	0.02	1.07	4.56	2.16	0.03	1.01	4.40
New LBBB	1.12	0.73	0.58	2.15				

Abbreviations: AV, aortic valve; CAD, coronary artery disease; ESRD, end-stage renal disease; HAVB, high-grade atrioventricular block; LBBB, left bundle branch block; LVEF, left ventricular ejection fraction; NYHA, New York Heart Association; OR, odds ratio; RBBB, right bundle branch block; TAVR, transcatheter aortic valve replacement; TIA, transient ischemic attack**.**

**Figure 2:**
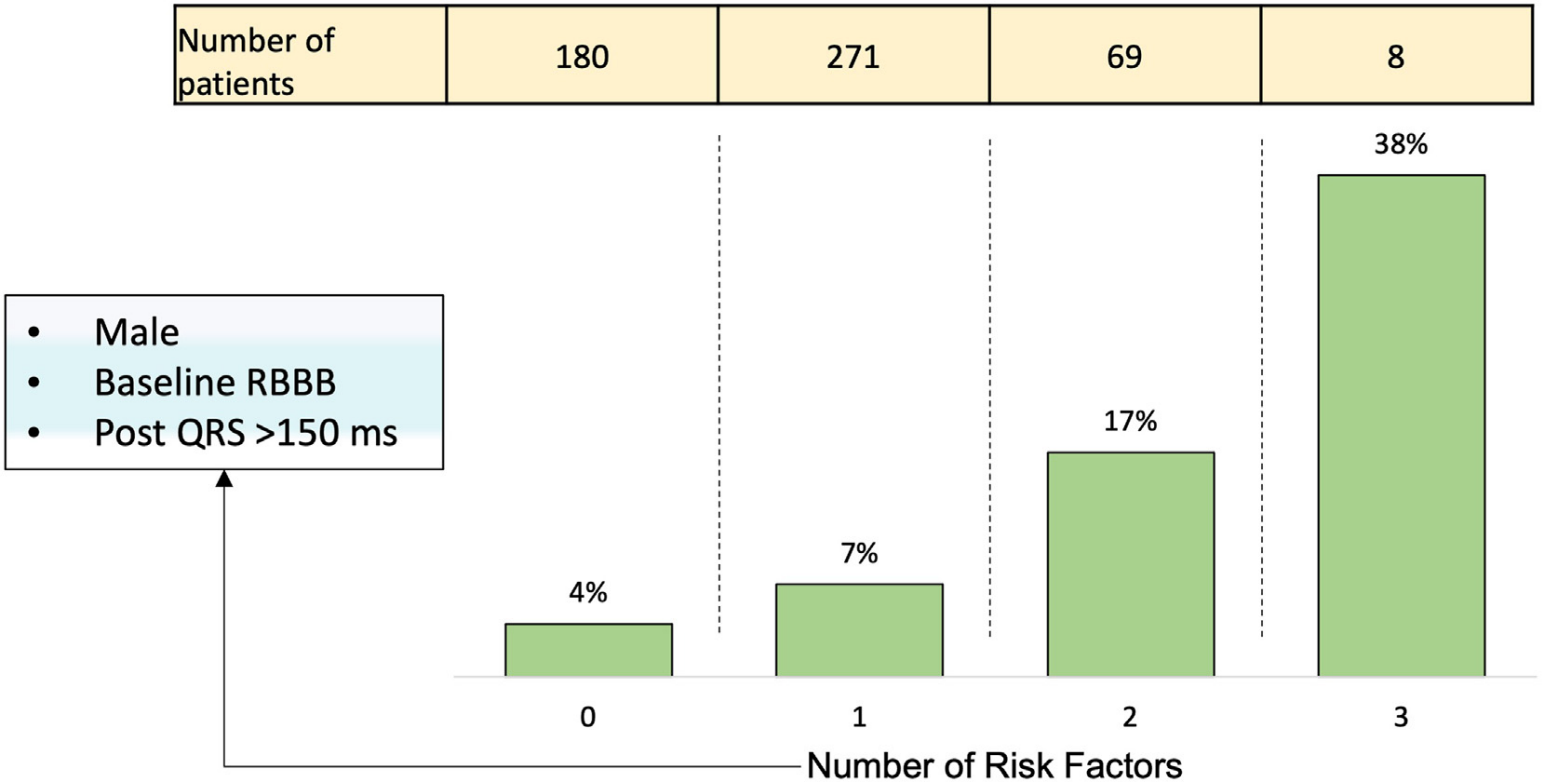
Probability of 30-day HAVB based on logistic regression results Abbreviations: HAVB, high-grade atrioventricular block; RBBB, right bundle branch block.

The increase in 30-day HAVB risk based on the number of risk factors was 4, 7, 17, and 38% (0, 1, 2, and 3 risk factors, respectively; Figure 2).

### Overall Survival and Predictors of All-Cause Mortality

Over a median follow-up of 2 years (1.3-2.7), the overall mortality rate in this cohort was 15.0%, with a 30-day mortality rate of 0.57% (n = 3). The hospital length of stay was 2 (1-2) days.

## Discussion

This study has the following novel findings: 1) rate of 30-day HAVB in patients on ambulatory cardiac monitor post-TAVR varies according to conduction system disease on postprocedure 12 lead ECG; 2) 30-day HAVB was more common in male patients; 3) 80.5% of HAVB events occurred during the first 2 ​weeks post-TAVR; 4) the majority of HAVB events were asymptomatic (68%), and mortality resulting from delayed HAVB was rare; 5) predictors of 30-day HAVB included male sex, baseline RBBB when another concomitant conduction abnormality develops (such as PR prolongation or left fascicular block) post-TAVR, and post-TAVR QRS >150 ​ms. Importantly, 30-day and longer-term survival in this cohort was high, excluding any confounding from survivorship bias. The findings of this study suggest that the current algorithm used at our institution appropriately stratifies patients who are at low risk for development of HAVB and can be discharged without further monitoring. Additionally, given that the majority of HAVB events occurred during the first 2 ​weeks of monitoring, it may be reasonable to limit ambulatory monitoring to a 2-week duration in many instances.

Patients in group 2 represent a more challenging group to manage with an intermediate observed risk of HAVB that also displayed heterogeneity in rates of HAVB among different conduction patterns. For instance, patients with transient junctional rhythm were observed to have HAVB of 20%, whereas most other conduction patterns in this group (isolated PR > 240 ​ms, new isolated LBBB <150 ​ms, and nonspecific intraventricular conduction defect >120 ​ms) had HAVB rates of 6% to 7%. Although these rates may not be considered “high,” they are greater than the rates observed in group 1 patients. Ambulatory monitoring has the advantage of allowing for prompt hospital dismissal and identification of the minority of patients in this group that may develop HAVB who may benefit from a PPM. Importantly, patients with isolated baseline RBBB had a low rate of HAVB if no other new conduction abnormalities developed on post-TAVR ECG, confirming that this group of patients can be safely dismissed from the hospital with ambulatory monitoring.

After TAVR, rates of delayed HAVB are estimated to range between 3.7% and 10%,^4^^,^^5^^,^^7^ in studies comprising smaller patient sample sizes (ranging between 118 and 219) on ambulatory monitoring. The rate of HAVB can be as high as 15% in patients with new LBBB discharged on 12-month ambulatory monitoring.^10^ An important factor worth noting is that delayed HAVB was defined differently in these prior studies (>24 vs. >48 ​hours). In the present study, all patients who were discharged on ambulatory cardiac monitoring were included without being restricted to a specific time cutoff. Importantly, a graded risk of delayed HAVB was observed according to the conduction system disease group, with group 1 patients having the lowest risk (5.0%), group 2 having an intermediate risk (6.2%), and group 3 having the highest risk (21.7%). The low rates of delayed HAVB in groups 1 and 2 suggest that a risk stratification algorithm based on the post-TAVR 12-lead ECG has a high negative predictive value for delayed HAVB and can effectively identify a group of patients appropriate for early dismissal from the hospital.

Risk factors for 30-day HAVB in the present study included male sex, baseline RBBB, and post-TAVR QRS >150 ​ms. These predictors are similar to other studies including Tian et al.^4^ (baseline RBBB OR = 6.86; [95% CI, 2.93-16.06]; *p* < 0.001), Ream et al.^5^ (baseline RBBB OR = 45.1; [95% CI, 5.17-393.4]; *p* < 0.001), and El-Sabawi et al.^7^ (baseline RBBB OR = 13.16; [95% CI, 8.32-20.83]; *p* < 0.001). In our study, male sex was also an independent risk factor for HAVB development, potentially due to greater comorbidities in male patients, with higher rates/duration of CAD, larger valve diameter, longer baseline PR and QRS intervals, and post-TAVR PR interval (*p* < 0.001; Table 4). Post-TAVR QRS of greater than 150 was also found to be a predictor of HAVB development. Similarly, new LBBB post-TAVR was found to be an independent predictor of late HAVB development in El-Sabawi et al.^7^ report (OR = 2.36; [95% CI, 2.36-13.23]; *p* < 0.001. These risk factors, in addition to the length of membranous septum,^11^ are all important to consider when choosing and counseling patients for TAVR and for the management of its conduction disease thereafter.

The results of this study demonstrate relatively low rates of 30-day HAVB percentage requiring PPM implantation using a contemporary conduction management algorithm and suggest that the current approach used for detection of HAVB post-TAVR can safely rule out the need for prophylactic PPM implantation. On the other hand, these data suggest that 30-day monitoring may be unnecessary in some patients, and future studies to examine the outcomes of a more conservative 30-day monitoring strategy are needed. Likewise, future studies should assess whether prophylactic PPM implantation in higher conduction system disease groups can be avoided. Although the 30-day HAVB from monitored patients were less than 6.5% in groups 1 and 2, asymptomatic events were more common than symptomatic, highlighting the challenge of identifying HAVB events without the ambulatory monitoring. Furthermore, some HAVB events may be benign and distinguishing which may warrant aggressive therapy requires further study. It remains crucial to evaluate patients’ conduction disease risk on a case-by-case approach.

## Limitations

Limitations of this study include its retrospective observational nature. Moreover, the relatively low event number of 30-day HAVB precludes the development of a risk calculator to avoid model overfitting. The 3-group algorithm used in this analysis was implemented clinically at our institution in April 2020, which likely accounts for variability in management between conduction risk groups in this analysis, which included patients from 2016 to 2021. While the accuracy of our model was high, the area under the ROC curve was acceptable. This establishment of a comprehensive risk score remains challenging due to the rarity of events and the prophylactic use of PPM in group 3 patients (precluding us from knowing whether these criteria are all high-risk or not). Lastly, the majority of this cohort received BEVs, and these data may not apply to predominant SEV implantation practices.

## Conclusions

The overall 30-day HAVB rate was 7.8% in patients discharged on ambulatory monitoring post-TAVR, with lower rates of HAVB observed in groups with less conduction system disease on 12-lead ECG. The majority of HAVB events occurred in the first 2 ​weeks post-TAVR, and the majority of patients with HAVB were asymptomatic. Predictors of 30-day HAVB included male sex, baseline RBBB (in the presence of another conduction abnormality), and post-TAVR QRS >150 ​ms. A risk stratification algorithm based on the post-TAVR 12-lead ECG has a high negative predictive value for HAVB development, can identify patients appropriate for early dismissal from the hospital, and may allow for lower rates of PPM implantation for prophylactic purposes. Future studies should evaluate whether risk stratification models correlate with long-term mortality in these patients.

## Ethics Statement

The Mayo Clinic Institutional Review Board approved this project.

## Funding

The authors have no funding to report.

## Disclosure Statement

The authors report no conflict of interest.
